# Novel Interplay Between Smad1 and Smad3 Phosphorylation via AGE Regulates the Progression of Diabetic Nephropathy

**DOI:** 10.1038/s41598-018-28439-1

**Published:** 2018-07-12

**Authors:** Hiroyuki Ono, Hideharu Abe, Akiko Sakurai, Arisa Ochi, Tatsuya Tominaga, Masanori Tamaki, Seiji Kishi, Taichi Murakami, Kojiro Nagai, Masayuki Kohashi, Toshio Doi

**Affiliations:** 1 Department of Nephrology, Institute of Biomedical Sciences, Tokushima University Graduate School, Tokushima, Japan; 2 Biology and Translational Research Unit, Department of Medical Innovations, New Drug Research Division, Otsuka Pharmaceutical. Co. Ltd., Tokushima, Japan

## Abstract

Diabetic nephropathy (DN) is the major cause of end-stage renal failure and is associated with increased morbidity and mortality compared with other causes of renal diseases. We previously found that Smad1 plays a critical role in the development of DN both *in vitro* and *in vivo*. However, functional interaction between Smad1 and Smad3 signaling in DN is unclear. Here, we addressed the molecular interplay between Smad1 and Smad3 signaling under a diabetic condition by using *Smad3*-knockout diabetic mice. Extracellular matrix (ECM) protein overexpression and Smad1 activation were observed in the glomeruli of db/db mice but were suppressed in the glomeruli of *Smad3*^+/−^; db/db mice. Smad3 activation enhanced the phosphorylation of Smad1 C-terminal domain but decreased the phosphorylation of linker domain, thus regulating Smad1 activation in advanced glycation end product-treated mesangial cells (MCs). However, forced phosphorylation of the Smad1 linker domain did not affect Smad3 activation in MCs. Phosphorylation of the Smad1 linker domain increased in *Smad3*^+/−^; db/db mice and probucol-treated db/db mice, which was consistent with the attenuation of ECM overproduction. These results indicate that Smad3 expression and activation or probucol treatment alters Smad1 phosphorylation, thus suggesting new molecular mechanisms underlying DN development and progression.

## Introduction

Diabetic nephropathy (DN) is a life-threatening complication of diabetes mellitus and is now the major cause of end-stage kidney disease worldwide^1^. Therefore, it is important to investigate the pathogenesis of DN and establish effective therapies for its treatment. Proteinuria and progressive renal insufficiency are the characteristic clinical manifestations of DN. Structurally, DN is characterized by mesangial matrix expansion caused by the excessive deposition of extracellular matrix (ECM) proteins in the mesangial area^2^. Excessive synthesis of ECM proteins (types I, III, and IV collagens [Col1, Col3, and Col4, respectively]) by mesangial cells (MCs) promotes the development of glomerular sclerosis with renal dysfunction^3–5^. Multiple factors are involved in the pathogenesis of DN. Advanced glycation end products (AGEs) produced as a result of hyperglycemia stimulate the production of ECM proteins^6–8^. In MCs, AGEs induce activation of various signaling pathways. In particular, transforming growth factor-β (TGF-β) superfamily, the main driving force in the development of glomerulosclerosis, is suggested to stimulate ECM protein deposition by MCs^9^. TGF-β signaling is an important pathway underlying DN development^10–13^. However, the precise role of Smad3 signaling pathway activation under diabetic conditions is not completely understood.

We previously showed that Smad1 transcriptionally regulates the expression of Col4, a major component of excessive mesangial ECM protein deposition in DN, and other ECM proteins such as Col1 and Col3^14,15^. Smad1 is an intracellular molecule that was originally detected as a signal transducer of the TGF-β superfamily^16^. These stimuli induce the phosphorylation of Smad1 C-terminal domain^17^, its interaction with Smad4, and its translocation into the nucleus where it regulates the transcription of specific target genes^18^. Thus, Smad1 is the key signaling molecule directly involved in the initiation and progression of glomerulosclerosis in DN and other kidney diseases^19,20^. However, the correlation between Smad1 and Smad3 signaling is unclear.

Smad1 contains two major domains, namely, N-terminal MH1 domain and C-terminal MH2 domain, that are connected by a linker domain. The MH1 domain binds to DNA, whereas the MH2 domain binds to membrane receptors to activate nucleoporins for nuclear translocation and other Smad proteins and nuclear factors to form transcriptional complexes^17^. Some studies indicate the phosphorylation of Smad1 linker domain prevents the nuclear translocation of Smad1, thus inactivating Smad1 signaling in Xenopus embryogenesis^21^ and mouse stem cells^22^. Moreover, Ser206 is the most prominently phosphorylated site in linker region, and we generated Smad1 (S206E) as an active form of phosphorylated Smad1 at the linker domain^22^. However, no study performed to date has assessed regulatory mechanisms underlying the significance of the phosphorylation of the Smad1 linker domain in diabetes mellitus.

In the present study, we determined molecular mechanisms underlying the interplay between Smad1 and Smad3 signaling under a diabetic condition by using *Smad3*-knockout diabetic mice. Phosphorylation of the Smad1 linker domain, which was partially regulated by Smad3 signaling, attenuated glomerulosclerosis, suggesting that Smad1 linker domain can be used as a target for treating DN. Some drugs are effective for treating DN; however, none of the known drugs target the Smad1 linker domain. Therefore, in the present study, we evaluated the effect of antioxidant probucol, which ameliorates glomerulosclerosis^23^, on the phosphorylation of the Smad1 linker domain.

## Results

### Reduced Smad3 Activity Improves DN in Mice

**Figure 1:**
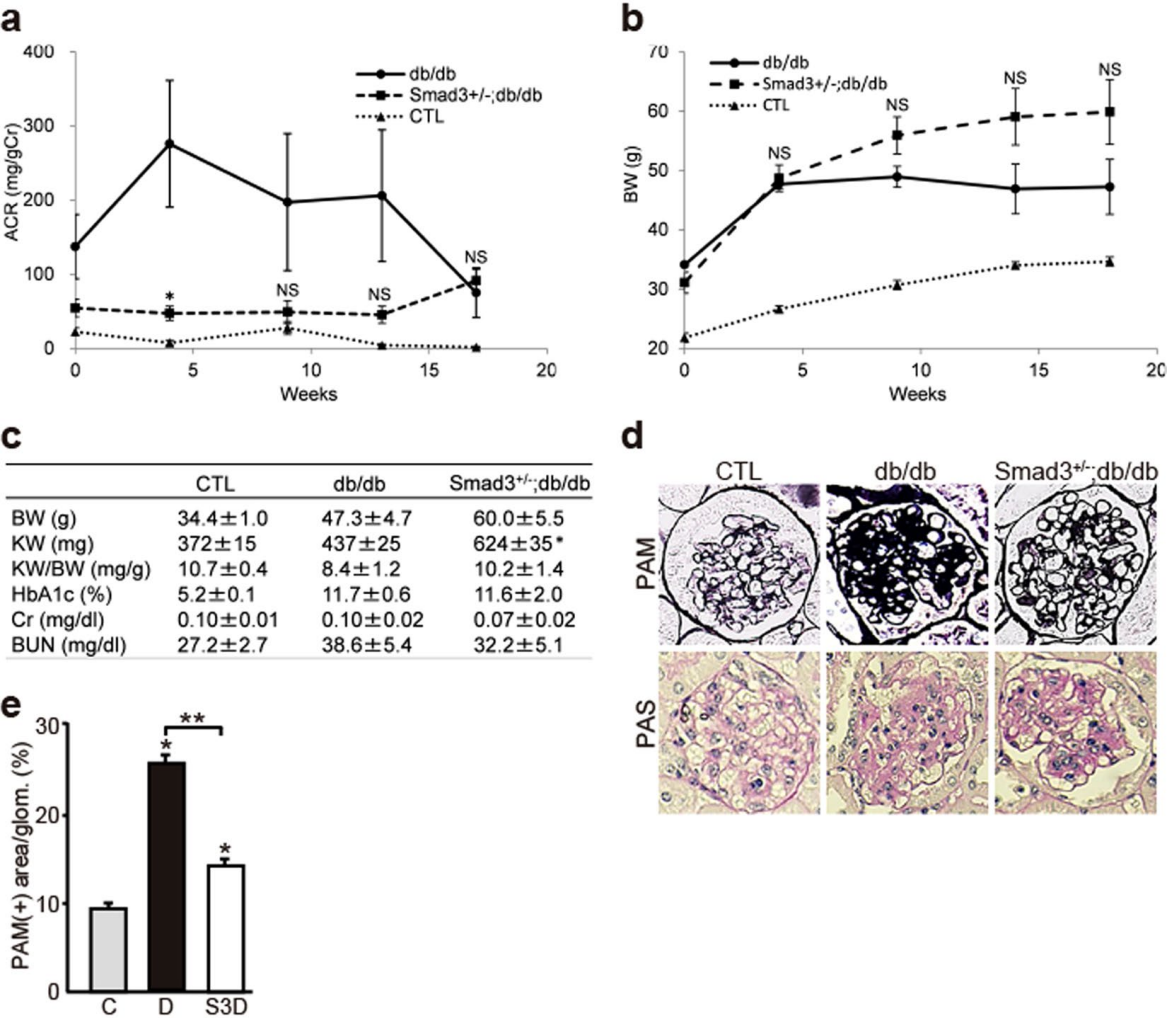
Effect of Smad3 deletion on phenotypic changes in diabetic nephropathy. (**a**) Time course of changes in urinary albumin excretion (as the ratio of albumin to creatinine) in the three groups – non-diabetic mice (normal control (CTL) mice) (▴), diabetic mice (db/db mice) (●) and *Smad3*-knockout diabetic mice (*Smad3*^+/−^; db/db mice) (▪) (n = 10 for normal control mice; n = 10 for db/db mice; n = 5 for *Smad3*^+/−^; db/db mice; NS, not significant, *p < 0.05 versus db/db mice, t test). (**b**) Time course of changes in body weight in the above three groups (NS, not significant, *p < 0.05 versus db/db mice, t test). (**c**) Biochemical data of the above groups (results are expressed as the mean ± S.E., *p < 0.05 versus db/db mice). (**d**) Representative photomicrographs of PAM staining from normal control mice (left panels), db/db mice (middle panels), and *Smad3*^+/−^; db/db mice (right panels). (**e**) Mesangial sclerotic fraction in the above three groups was determined as percentage of mesangial matrix area per total glomerular surface area. All glomeruli were analyzed for each sample (*p < 0.001 versus normal control mice, **p < 0.001 versus db/db mice, t test). C, D, and S3D stand for normal control mice, db/db mice, and *Smad3*^+/−^; db/db mice, respectively.

**Figure 2:**
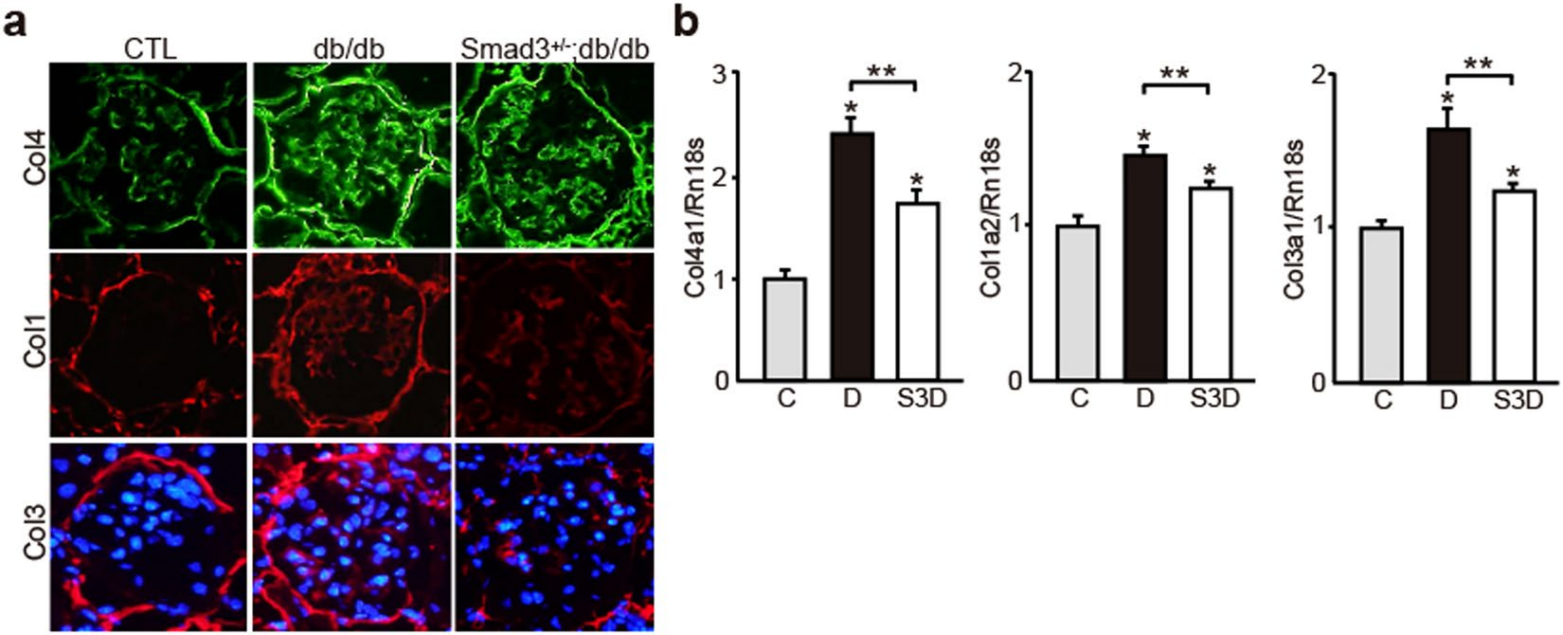
Glomerular expression of ECM proteins in diabetic Smad3 knockout mice and control mice. (**a**) Representative photomicrographs of immunohistochemical staining of ECM proteins (Col4, Col1 and Col3) in the three groups (n = 10 for normal control mice; n = 10 for db/db mice; n = 5 for *Smad3*^+/−^; db/db mice; *p < 0.05 versus normal control mice, **p < 0.05 versus db/db mice, t test). Nuclei were stained with DAPI (blue). (**b**) The expression level of Col4 (Col4a1), Col1 (Col1a2), and Col3 (Col3a1) mRNA in the glomeruli in the above three groups. They were analyzed by qPCR and normalized to the expression of Rn18s. The values are expressed as the mean ± S.E. (*p < 0.05 versus normal control mice, **p < 0.05 versus db/db mice, t test). C, D, and S3D stand for normal control mice, db/db mice, and *Smad3*^+/−^; db/db mice, respectively.

The glomeruli of normal mice lacked Smad1 expression but showed steady Smad3 expression. *Smad3*^−/−^ mice are viable but look feeble at birth and show various defects such as impaired immune function, growth retardation, decreased survival, and small body size compared with *Smad3*^+/−^ littermates^24^. Therefore, in the present study, we assessed *Smad3*^+/−^ mice for a comparatively long period to examine the effects of Smad3 phosphorylation and to evaluate the molecular interaction between Smad3 and Smad1 in DN. The degree of albuminuria was lower in *Smad3*^+/−^; db/db mice than in db/db mice during the experimental period (Fig. 1a). Diabetes-associated reduction in body weight was not shown in *Smad3*^+/−^; db/db mice (Fig. 1b,c). Serum creatinine (Cr), blood urea nitrogen (BUN), and HbA1c levels were not different between *Smad3*^+/−^; db/db mice and db/db mice; however, the kidney weight of db/db mice was lower than that of *Smad3*^+/−^; db/db mice (Fig. 1c). We performed histological examination with periodic acid Schiff (PAS) and periodic acid methenamine silver (PAM). Histologically, most glomeruli of db/db mice showed widespread increases in PAS- or PAM-positive mesangial areas, which were suppressed in *Smad3*^+/−^; db/db mice (Fig. 1d). Moreover, expansion of the PAM-positive mesangial areas was significantly attenuated in *Smad3*^+/−^; db/db mice compared with that in db/db mice (Fig. 1e). We next examined the expression of ECM proteins such as Col4, Col1, and Col3. Expression of these proteins was higher in db/db mice than in control mice but was lower in *Smad3*^+/−^; db/db mice than in db/db mice (Fig. 2a). We next performed qPCR by using mRNA isolated from the renal cortex of mice in the different groups and evaluated the mRNA expression levels of *Col4*, *Col1*, and *Col3*. Expression of these collagen genes increased in db/db mice but was suppressed in *Smad3*^+/−^; db/db mice (Fig. 2b). These data suggest that repression of *Smad3* expression in the diabetic glomeruli attenuates DN progression.

Many previous reports have demonstrated that AGE-RAGE (receptor for AGE) plays a critical role for the progression of DN using db/db mice as the model^25^. In addition, TGF-β signaling pathway is activated at the downstream of AGE-RAGE signaling pathway as previously reported by other groups and our own data^26–28^. Therefore, we first examined which TGF-β isoform, TGF-β1-3, is expressed and involved in diabetic *Smad3*- knockout kidneys. TGF-β1 expression was markedly up-regulated in diabetic control mice. Similar upregulation was found in diabetic *Smad3*-knockout mice. In contrast, alteration of TGF-β2 and TGF-β3 expressions was not observed (Supplementary Fig. S1). Moreover, expression level of TGF-β1 was relatively enhanced in *Smad3*-knockout mice in each group. Next, we measured the AGE levels in serum and kidney in each group and examined the activation of AGE-RAGE-NFκB signaling pathway. Compared with non-diabetic normal littermates, db/db mice exhibited increased AGE-product formation in serum and renal cortex proteins in both groups (control and *Smad3*^+/−^) (Supplementary Fig. S2a,b). Activation of the AGE/RAGE/NFκB pathway may play an important role in the pathogenesis of the DN^26,29^. To verify that AGE-RAGE signal activation, we assessed the expression and phosphorylation (activation) status of NFκB subunit p65. In diabetic mice, p-p65 expression was eminently induced, indicating that AGE-RAGE signal was activated in both groups (control and *Smad3*^+/−^) in a diabetic condition (Supplementary Fig. S2c).

**Figure 3:**
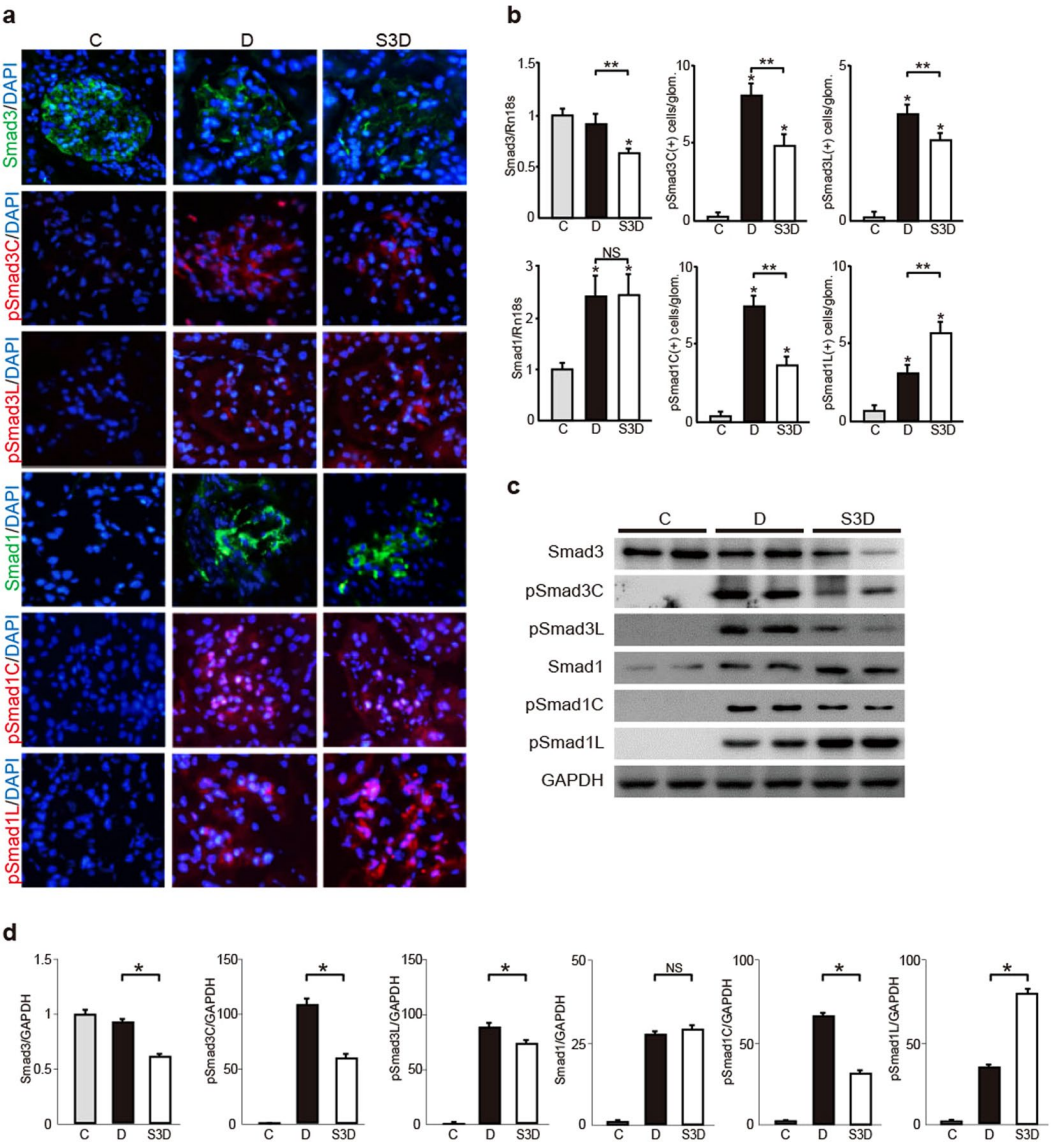
Altered expression of both Smad1 and Smad3 and their phosphorylation forms in diabetic mice. (**a**) Immunohistochemical staining of Smad1, pSmad1C, pSmad1L, Smad3, pSmad3C, and pSmad3L in glomeruli of three groups (normal control, db/db and *Smad3*^+/−^; db/db mice). (**b**) Quantitation of Smad1, pSmad1C, pSmad1L, Smad3, pSmad3C and pSmad3L expression in the above three groups. Smad1 and Smad3 were assessed by qPCR analysis, and normalized to the expression of Rn18s. As to pSmad1C, pSmad1L, pSmad3C and pSmad3L, the number of each phosphorylated Smad1 or Smad3 protein positive nuclei was counted, and the mean values (per one glomerulus) were calculated (results are expressed as the mean ± S.E., NS, not significant, *p < 0.05 versus normal control mice, **p < 0.05 versus db/db mice, t test). (**c**) Western blot analysis for Smads and their phosphorylated forms in whole kidney extracts from three groups. Each lane represents a representative lysates from each mouse. (**d**) Optical densitometry of these proteins in western blot was shown. The values are expressed as the mean ± S.E. (NS, not significant, *p < 0.05, t test). C, D, and S3D stand for normal control mice, db/db mice, and *Smad3*^+/−^; db/db mice, respectively. C, D, and S3D stand for normal control mice, db/db mice, and *Smad3*^+/−^; db/db mice, respectively. Uncropped scans are presented in Supplementary Fig. S6.

Next, we performed immunohistochemical analysis to clarify the involvement of Smad1 and Smad3 phosphorylation in DN progression. Control mice contained negligible levels of Smad1, phosphorylated Smad1 (pSmad1C and pSmad1L), and phosphorylated Smad3 (pSmad3C and pSmad3L) (Fig. 3a). We previously reported that Smad1 is induced and subsequently phosphorylated at its C-terminal domain in the diabetic glomeruli. Consistently, we observed the induction and phosphorylation of the Smad1 C-terminal domain in the glomeruli of db/db mice in the present study. Furthermore, we detected the phosphorylation of Smad1 linker domain (Fig. 3a). The level of pSmad1C and pSmad1L decreased and increased, respectively, in *Smad3*^+/−^; db/db mice compared with that in db/db mice (Fig. 3b–d). Next, we investigated the phosphorylation of the Smad3 C-terminal and linker domains in db/db mice. Smad3 expression decreased in the glomeruli of *Smad3*^+/−^; db/db mice; however, no significant difference in Smad3 expression was observed between control and db/db mice. However, phosphorylation of the Smad3 C-terminal and linker domains was attenuated in *Smad3*^+/−^; db/db mice, compared with that in db/db mice (Fig. 3a–d). These data suggest that altered Smad1 phosphorylation in relation to Smad3 expression in the diabetic glomeruli plays important roles in DN progression.

### AGE stimulation significantly activates Smad1 and Smad3 in MCs

To compare the effects of exposure of high glucose and AGE on MCs, we examined the expression and phosphorylation of Smad1 and Smad3 by western blot analysis. Similar to the previous our examination^14^, AGE stimulation showed significant activation of Smad1 and Smad3 in MCs, compared with high glucose stimulation (Supplementary Fig. S4).

### Smad1 Phosphorylation Increases in Smad3-null MCs

**Figure 4:**
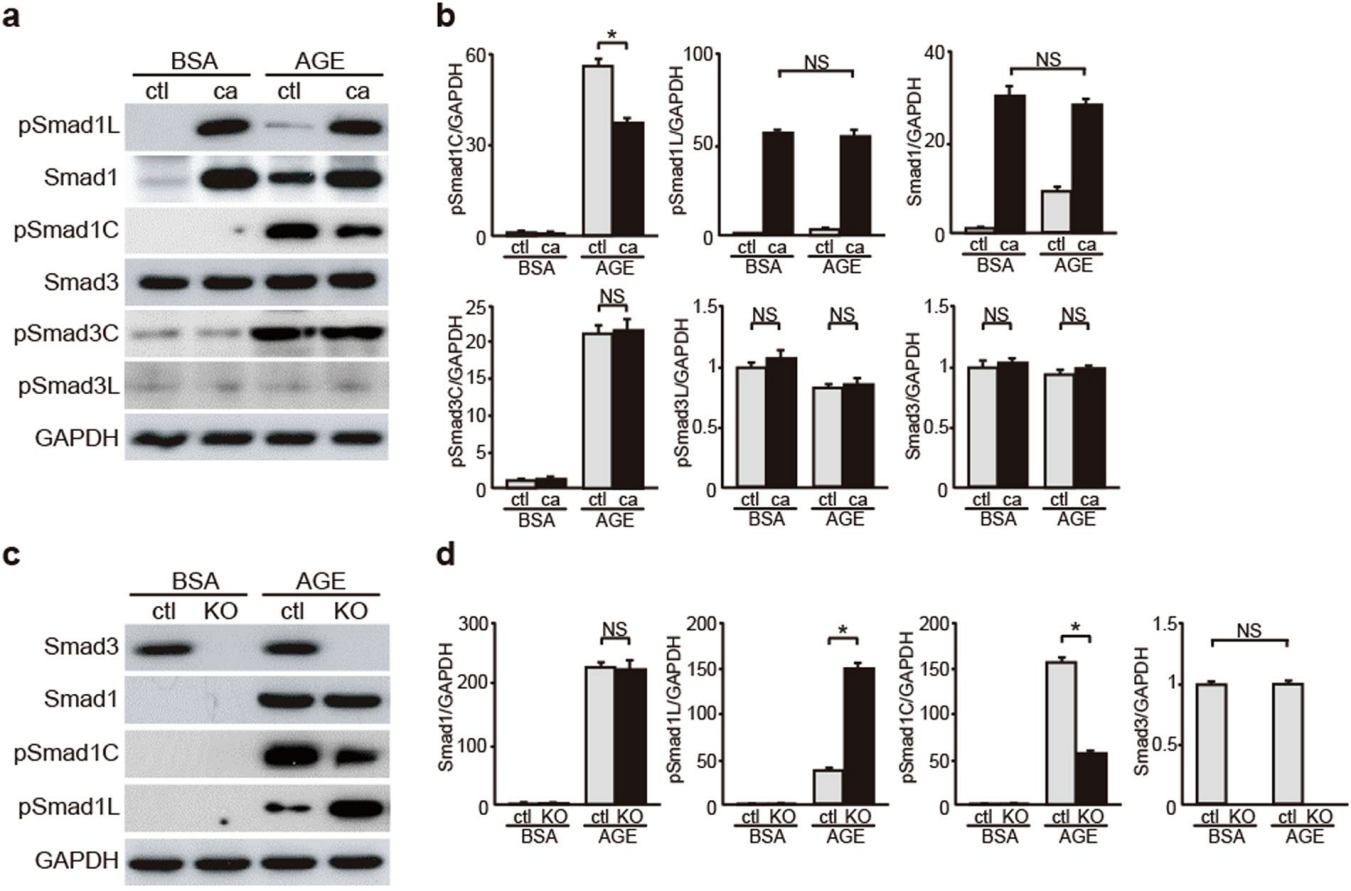
Evaluation of interactive roles for Smad1 and Smad3 activation in MCs in a diabetic condition. (**a**) Effects of overexpression of constitutively active form of Smad1 carrying a mutation in the linker domain (ca) on the modulation in Smad1 and Smad3 signaling pathways in BSA- or AGE-treated MCs. Phosphorylation of Smad1 and Smad3 proteins in MCs were monitored by western blotting. After a 48-h exposure to AGE or BSA (5 μg/ml), equal amounts of cell lysates were subjected to western blotting. GAPDH was used as a loading control. One of three independent experiments is shown. ctl, empty vector; ca, Smad1 carrying S206E mutation. (**b**) Optical densitometry of these proteins in western blot was shown. The values are expressed as the mean ± S.E. (NS, not significant, *p < 0.05, t test). (**c**) Effects of Smad3 deletion on the modulation in Smad1 and Smad3 signaling pathways in BSA- or AGE-treated *Smad3*-null MCs (KO). After a 48-h exposure to AGE or BSA (5 μg/ml), equal amounts of cell lysates were subjected to western blotting. GAPDH was used as a loading control. One of three independent experiments is shown. (**d**) Optical densitometry of these proteins in western blot was shown. The values are expressed as the mean ± S.E. (NS, not significant, *p < 0.05, t test). Uncropped scans are presented in Supplementary Fig. S7.

To clarify the molecular interaction between Smad1 and Smad3 under a diabetic condition, we examined whether Smad1 signaling was associated with Smad3 signaling in cultured AGE-treated MCs. In AGE-treated MCs, Smad1 expression remarkably increases, though the steady state expression level of Smad1 is negligible^14^. Moreover, Smad1 and Smad3 are activated through the phosphorylation of their C-terminal domains^14,30^. Meanwhile, the phosphorylation of Smad1 linker domain prevents the nuclear translocation of Smad1, thus inactivating Smad1 signaling^21^. Similar to Smad1 liker phosphorylation, phosphorylation of Smad3 linker domain inhibits Smad3 nuclear accumulation^18,31^. We first carried out overexpression experiments by transfecting four different constructs (empty vector, wild-type Smad1, Smad1 (S206E), and Smad1 (S206A)) into MCs, and confirmed that S206E was actually constitutively more active, by western blot analysis (Supplementary Fig. S3). In the present study, pSmad1C level significantly decreased and Smad3, pSmad3C, and pSmad3L levels remained unchanged in AGE-treated MCs overexpressing pSmad1L (Fig. 4a,b), indicating that Smad1 linker domain activation suppressed Smad1 activation without affecting Smad3 expression and activation under diabetic conditions. In contrast, pSmad1C level significantly decreased and pSmad1L level significantly increased in AGE-treated *Smad3*-null MCs (Fig. 4c,d). Together, these results suggest that Smad3 expression and activation exert an important effect on Smad1 activation and subsequent ECM protein overexpression in DN.

### Constitutively Activated Smad3 Regulates Smad1 Phosphorylation in AGE-Treated MCs

**Figure 5:**
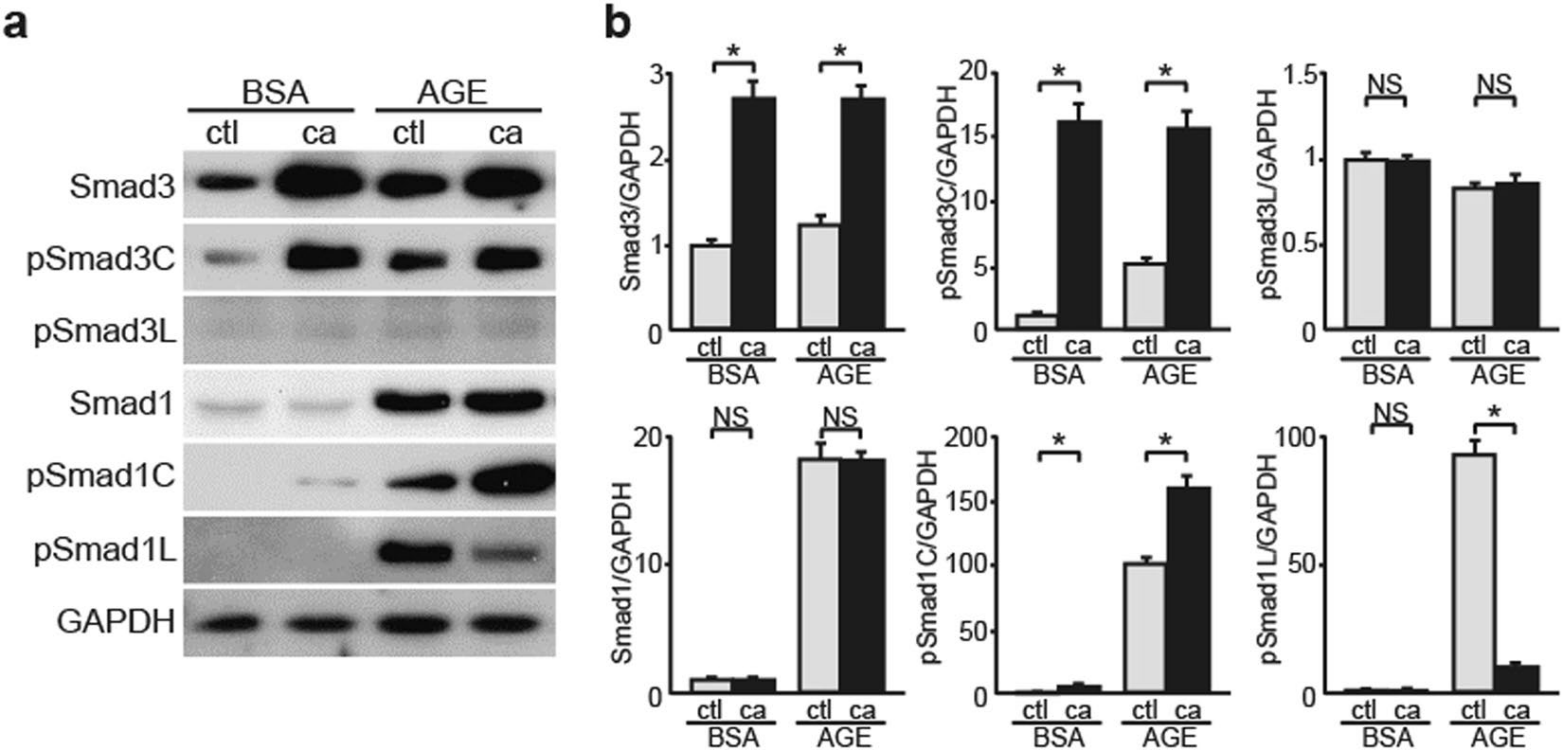
Influence of Smad3 on the balance of Smad1 C-terminal and linker phosphorylation. Effects of overexpression of constitutively active form of Smad3 (ca) on the modulation of Smad1 and Smad3 signaling pathways in BSA- or AGE-treated MCs. (**a**) After the transfection and successive 48 h of incubation, MCs were treated with BSA or AGE for 48 h. Equal amounts of cell lysates were subjected to western blotting. GAPDH was used as a loading control. Data are representative of at least three independent experiments. (**b**) Optical densitometry of these proteins in western blot was shown. The values are expressed as the mean ± S.E. (NS, not significant, *p < 0.05, t test). ctl, empty vector; ca, constitutively active form of Smad3. Uncropped scans are presented in Supplementary Fig. S8.

We next examined the effects of Smad3 activation on Smad1 signaling by analyzing constitutively activated Smad3 (caSmad3)-overexpressing MCs. CaSmad3 overexpression significantly increased pSmad1C level and decreased pSmad1L level without affecting Smad1 and pSmad3L expression in AGE-treated MCs (Fig. 5a,b). However, these effects were not observed in control BSA-treated MCs. These findings suggest that pSmad3C modulates the balance between pSmad1C and pSmad1L and its subsequent activation under a diabetic condition. In addition, these results suggest that AGE-treated MCs contain some unknown factors that mediate Smad3 signaling to activate Smad1 signaling.

### Probucol Enhances the Phosphorylation of Smad1 Linker Domain in MCs

**Figure 6:**
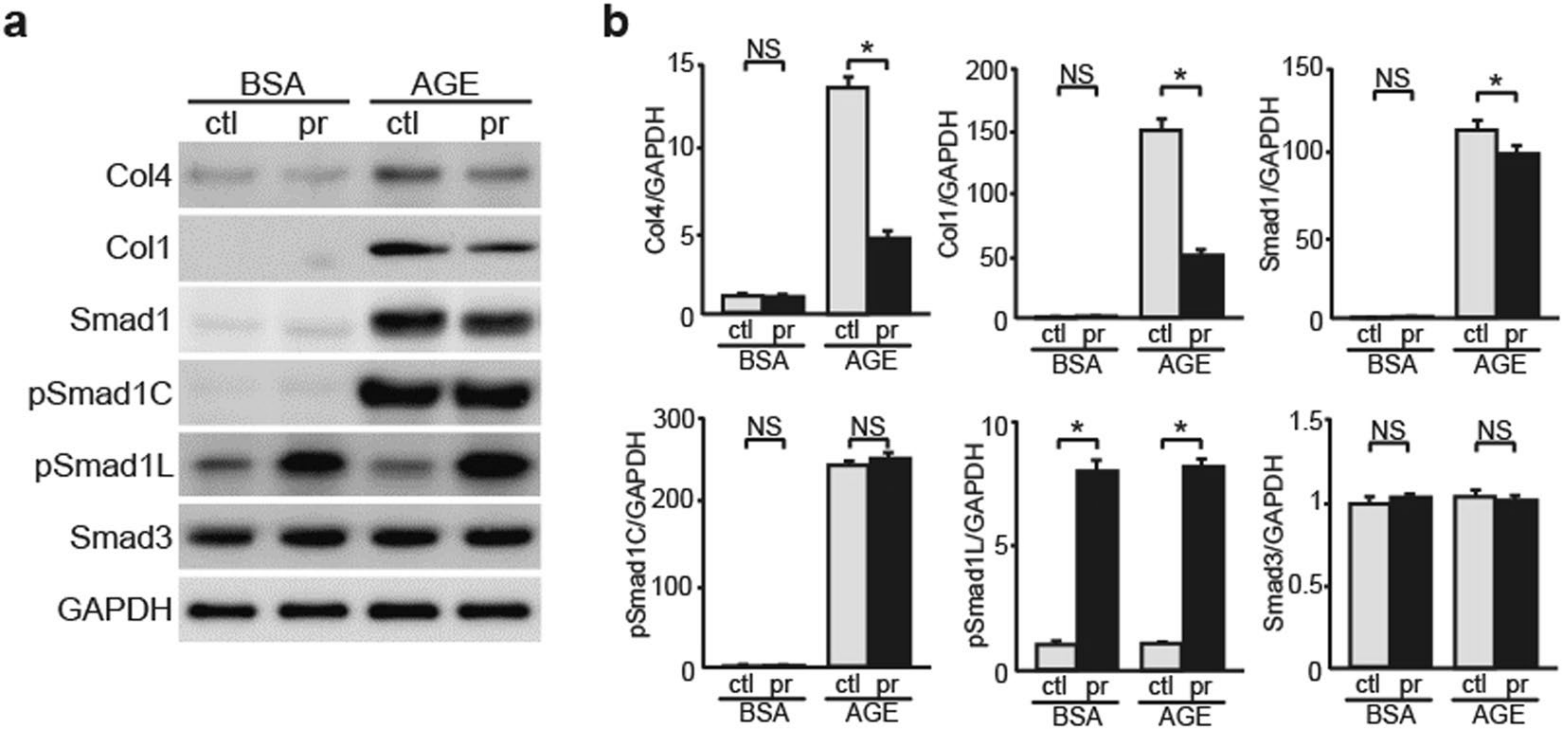
Effects of probucol on the modulation of Smad1 phosphorylation and ECM proteins expression in a diabetic condition. Thirty μM probucol or DMSO were added to MCs which were pre-exposed to BSA or AGEs for 36 h, and incubated for additional 12 h at 37 °C. (**a**) Equal amounts of cell lysates were subjected to western blot. GAPDH was used as a loading control. Data are representative of at least three independent experiments. (**b**) Optical densitometry of these proteins in western blot was shown. The values are expressed in mean ± S.E. (NS, not significant, *p < 0.05, t test). ctl and pr stand for DMSO- and probucol-treated MCs, respectively. Uncropped scans are presented in Supplementary Fig. S8.

Some studies indicate that antioxidant probucol delays DN progression^32,33^. However, target molecules that directly mediate this effect of probucol have not yet been identified. We examined the expression of Smad1 and glomerulosclerosis-related proteins in MCs treated with or without probucol. Probucol treatment showed increased pSmad1L level and decreased Smad1, Col4, and Col1 level in AGE-exposed MCs. In contrast, Smad1, Col4, and Col1 levels were not different between BSA-exposed MCs treated with or without probucol; however, pSmad1L level increased in BSA-exposed MCs treated with probucol (Fig. 6a,b). These findings indicated that probucol exerts unique effects on Smad1 phosphorylation in MCs, which are independent of the type of stimulation (AGE or BSA).

### Probucol Exerts Additional Effects to Improve DN in Mice

**Figure 7:**
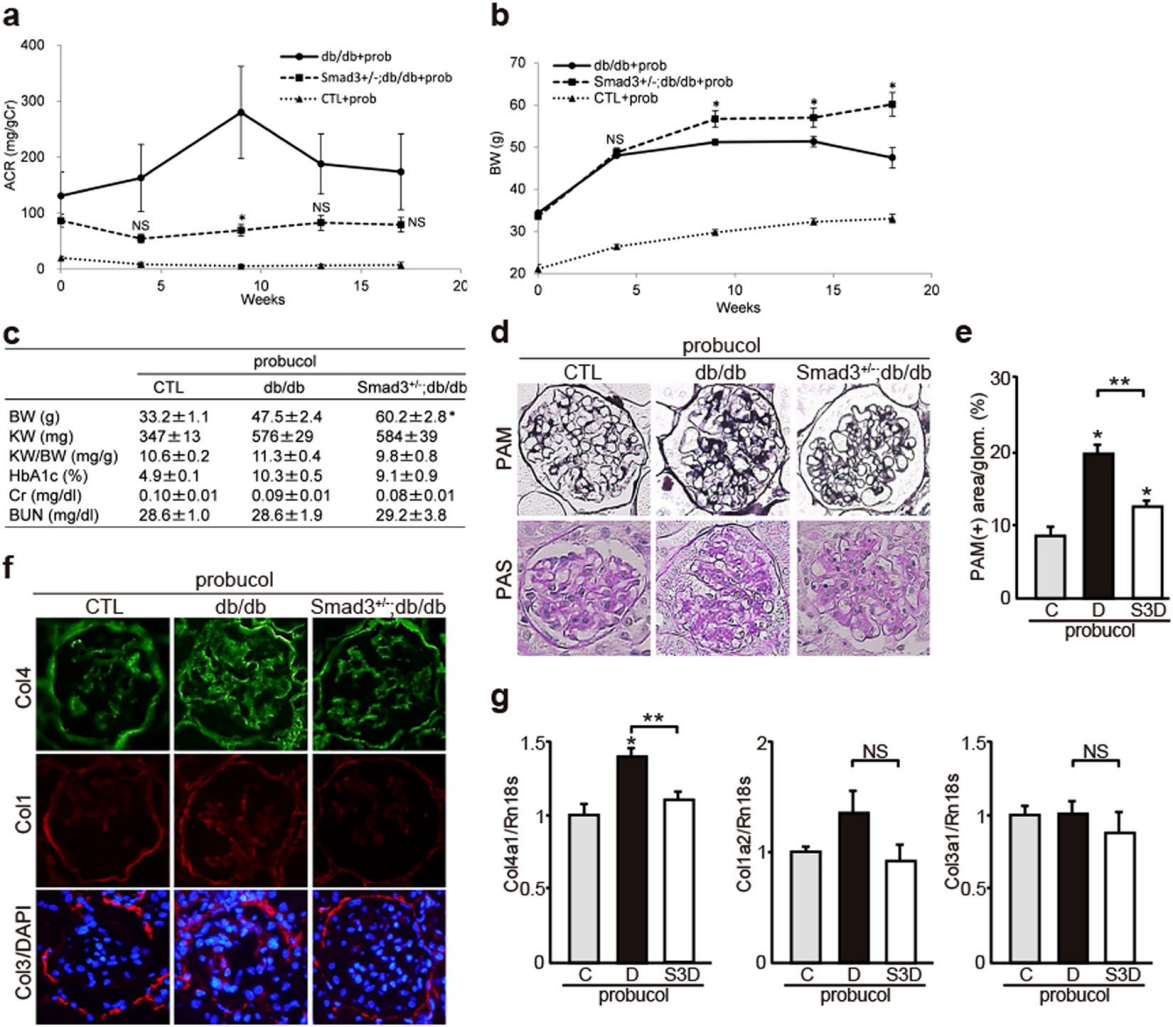
Effects of probucol on phenotypic changes in diabetic nephropathy with or without Smad3 deletion. (**a**) Time course of changes in urinary albumin excretion (as the ratio of albumin to creatinine) in the three groups treated with probucol - normal control (CTL) mice (▴), diabetic mice (db/db mice) (●), and *Smad3*-knockout diabetic mice (*Smad3*^+/−^; db/db mice) (▪) (n = 10 for normal control mice; n = 10 for db/db mice; n = 6 for *Smad3*^+/−^; db/db mice; NS, not significant, *p < 0.05 versus db/db mice, t test). (**b**) Time course of changes in body weight in the above three groups (NS, not significant, *p < 0.05 versus db/db mice, t test). (**c**) Biochemical data in the above three groups (results are expressed as the mean ± S.E., *p < 0.05 versus db/db mice). (**d**) Representative photomicrographs of PAS and PAM staining in the above three groups. (**e**) Mesangial sclerotic fraction in the above three groups was determined as percentage of mesangial matrix area per total glomerular surface area. All glomeruli were analyzed for each sample (*p < 0.01 versus probucol-treated normal control mice, **p < 0.01 versus probucol-treated db/db mice, t test). (**f**) Representative photomicrographs of immunohistochemical staining of ECM proteins (Col4, Col1 and Col3) in the above three groups. (**g**) The expression level of Col4, Col1, and Col3 in the glomeruli in the above three groups. They were analyzed by qPCR and normalized to the expression of Rn18s. The values are expressed as the mean ± S.E. (NS, not significant, *p < 0.05 versus probucol-treated normal control mice, **p < 0.05 versus probucol-treated db/db mice, t test). C, D, and S3D stand for normal control mice, db/db mice, and *Smad3*^+/−^; db/db mice, respectively.

**Figure 8:**
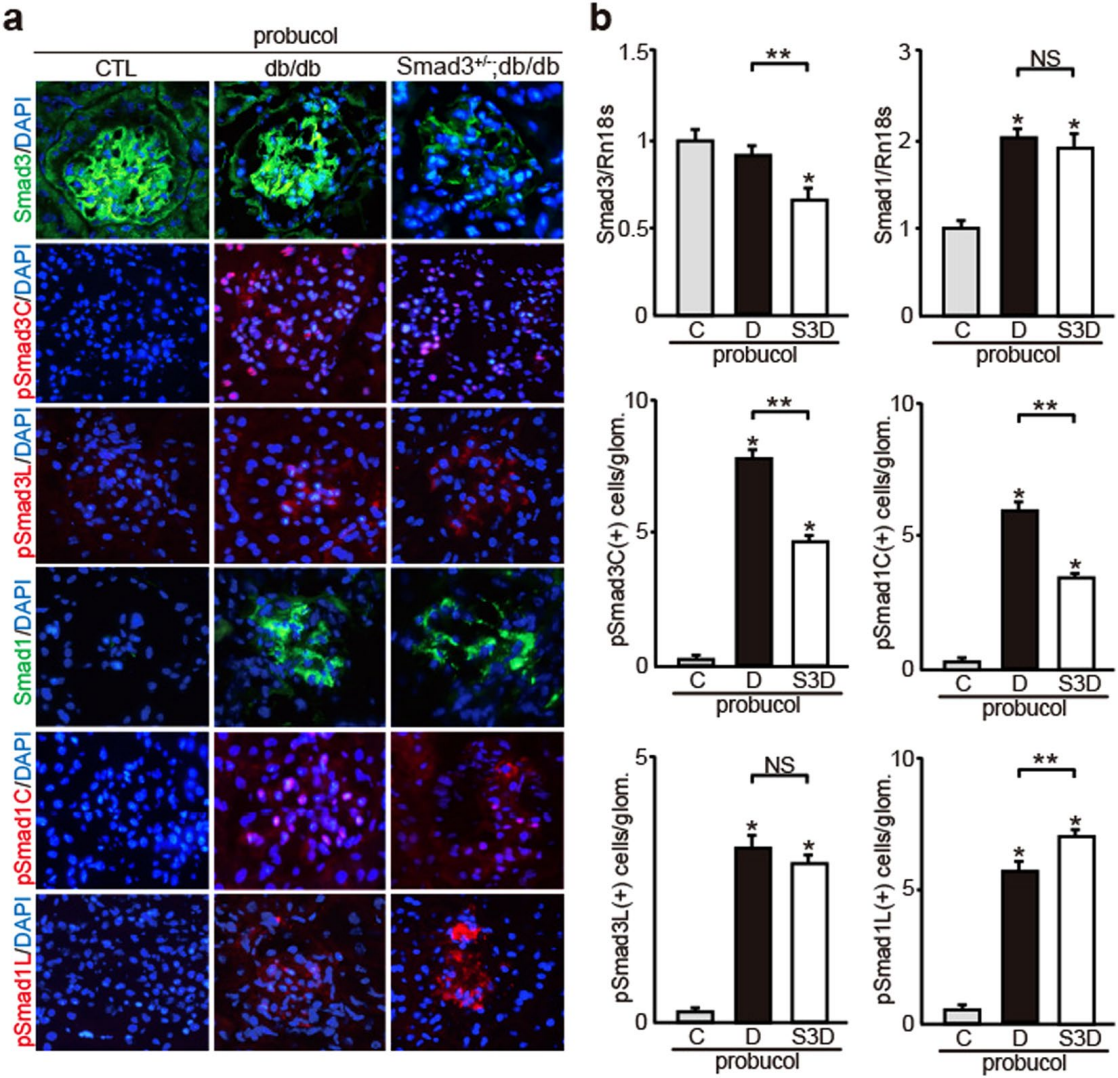
Effects of probucol on the changes of both Smad1 and Smad3 and their phosphorylation forms in diabetic mice. (**a**) Immunohistochemical staining of Smad1, pSmad1C, pSmad1L, Smad3, pSmad3C and pSmad3L in glomeruli of three groups treated with probucol (normal control, db/db and *Smad3*^+/−^; db/db mice). Each lane represents representative data. (**b**) Quantitation of Smad1, pSmad1C, pSmad1L, Smad3, pSmad3C, and pSmad3L expression in the above three groups. Smad1 and Smad3 were assessed by quantitative PCR analysis, and normalized to the expression of Rn18s. As to pSmad1C, pSmad1L, pSmad3C, and pSmad3L, the number of each phosphorylated Smad1 or Smad3 protein positive nuclei was counted, and the mean values (per one glomerulus) were calculated (results are expressed as the mean ± S.E., NS, not significant, *p < 0.05 versus probucol-treated normal control mice, **p < 0.05 versus probucol-treated db/db mice, t test). C, D, and S3D stand for normal control mice, db/db mice, and *Smad3*^+/−^; db/db mice, respectively.

To confirm the effect of probucol *in vivo*, we investigated whether probucol regulated the activation of Smad1 and/or Smad3 signaling in control, db/db, and *Smad3*^+/−^; db/db mice. The degree of albuminuria was significantly lower in probucol-treated *Smad3*^+/−^; db/db mice than in probucol-treated db/db mice (Fig. 7a). Diabetes-associated reduction in body weight was not shown in probucol-treated *Smad3*^+/−^; db/db mice (Fig. 7b,c). However, Cr, BUN, and HbA1c levels were not different between probucol-treated *Smad3*^+/−^; db/db mice and probucol-treated db/db mice (Fig. 7c). Moreover, T-Cho, LDL-Cho, HDL-Cho, and TG levels were not different between probucol-treated db/db mice and probucol-treated *Smad3*^+/−^; db/db mice. Histological analysis showed that expansion of PAM-positive mesangial areas (Fig. 7d,e) and overexpression of Col4 (Fig. 7f,g) were significantly inhibited in probucol-treated *Smad3*^+/−^; db/db mice compared with those in probucol-treated db/db mice. We next performed immunofluorescence analyses to clarify the contribution of Smad1 and Smad3 phosphorylation (Fig. 8a). Control mice showed negligible levels of Smad1, phosphorylated Smad1 (pSmad1C and pSmad1L), and phosphorylated Smad3 (pSmad3C and pSmad3L). Next, we confirmed the phosphorylation of Smad1 C-terminal and linker domains in the glomeruli of probucol-treated db/db mice. Phosphorylation of the Smad1 linker domain significantly increased (Fig. 8a,b) and that of the Smad1 C-terminal domain decreased (Fig. 8a,b) in the glomeruli of probucol-treated *Smad3*^+/−^; db/db mice. In addition, phosphorylation of the Smad3 C-terminal domain decreased in the glomeruli of probucol-treated *Smad3*^+/−^; db/db mice compared with that in probucol-treated db/db mice (Fig. 8a,b). However, no change in Smad1 expression level was observed between probucol-treated db/db mice and probucol-treated *Smad3*^+/−^; db/db mice (Fig. 8a,b).

Next, as to expansion of PAM-positive mesangial areas, ECM gene expressions, expressions of Smad1 and Smad3, and their phosphorylation, we made comparisons between untreated normal control mice and probucol-treated normal control mice, between untreated db/db mice and probucol-treated db/db mice, and between untreated *Smad3*^+/−^; db/db mice and probucol-treated *Smad3*^+/−^; db/db mice. In normal control mice, probucol treatment caused no change in PAM-positive mesangial areas, ECM gene expressions, expressions of Smad1 and Smad3, and their phosphorylation (Supplementary Fig. S5a). Meanwhile, Expansion of PAM-positive mesangial areas and ECM gene expressions significantly decreased in probucol-treated db/db compared with those in db/db mice (Supplementary Fig. S5b). Although Smad1 and Smad3 expression was not different between probucol-treated db/db mice and untreated db/db mice, pSmad1C and pSmad1L levels decreased and increased, respectively, in probucol-treated db/db mice compared with those in untreated db/db mice (Supplementary Fig. S5b). In addition, the phosphorylation of the Smad3 C-terminal and linker domains was not different between db/db mice and probucol-treated db/db mice (Supplementary Fig. S5b). Next, we compared untreated *Smad3*^+/−^; db/db mice and probucol-treated *Smad3*^+/−^; db/db mice with respect to the interactive effects between Smad3 expression level and probucol treatment. Expansion of PAM-positive mesangial areas and ECM gene expressions significantly decreased in probucol-treated *Smad3*^+/−^; db/db mice compared with those in untreated *Smad3*^+/−^; db/db mice (Supplementary Fig. S5c). Although Smad1 and Smad3 expression was not different between probucol-treated *Smad3*^+/−^; db/db mice and untreated *Smad3*^+/−^; db/db mice, pSmad1L level increased in probucol-treated *Smad3*^+/−^; db/db mice compared with that in untreated *Smad3*^+/−^; db/db mice. However, pSmad1C level was not different between probucol-treated *Smad3*^+/−^; db/db mice and untreated *Smad3*^+/−^; db/db mice. In addition, phosphorylation of the Smad3 C-terminal and linker domains was not different between untreated *Smad3*^+/−^; db/db mice and probucol-treated *Smad3*^+/−^; db/db mice (Supplementary Fig. S5c). These data indicate that probucol exerts similar effects on Smad1 phosphorylation as those observed in *Smad3*- knockout mice or MCs. Thus, these results suggest that probucol exerts additional effects on the phosphorylation of the Smad1 linker domain in a Smad3-independent manner.

## Discussion

DN is a severe progressing renal disease characterized by glomerular MCs proliferation and excessive ECM protein production^2^. TGF-β superfamily signaling pathway is significantly activated in the glomeruli of diabetic humans and animal models^34^. Previous studies have reported that TGF-β binds to types I and II TGF-β receptors on the cell membrane and induces Smad3 phosphorylation, thus promoting the synthesis of ECM proteins in DN, though Smad3 expression is unchanged in DN^30^. Thus far, TGF-β has been considered to be an important and critical mediator in the process of activation of Smad3^11^; however, the relationship between expression levels of three isoforms of TGF-β (TGF-β1, -β2, and -β3) and expression level of Smad3 has remained elusive. We previously found that TGF-β1 alone was up-regulated in STZ-induced diabetic mice^28^. Whereas three isoforms of TGF-β (TGF-β1, -β2, and -β3) was examined in db/db mice, TGF-β1 alone was up-regulated in diabetic conditions. In addition, TGF-β1 alone was up-regulated in *Smad3*- knockout mice. This may be because Smad3 mediates the growth inhibitory effect of TGF-β1 in many cell types. TGF-β1 expression level in *Smad3*-knockout mice was higher than that in control mice, which was observed in both groups (wild type and db/db mice). The upregulation of TGF-β1 expression levels might be caused by negative feedback regulation regarding the reduced Smad3 protein in MCs.

However, the precise role of Smad3 in the diabetic glomeruli, including transcriptional regulation of ECM proteins, and phosphorylation site in Smad3 are unclear. Because normal glomeruli exhibit steady-state Smad3 expression, we evaluated coincident changes in the phosphorylation of the Smad3 C-terminal and linker domains *in vivo*. To elucidate the functional role of Smad3 in DN progression, we conducted a long-term study by using genetically diabetic (db/db) mice. We observed that *Smad3*^+/−^; db/db mice showed partial improvement of albuminuria and significant decrease in ECM protein accumulation compared with db/db mice. Moreover, *Smad3*^+/−^; db/db mice did not show diabetes-associated reduction in body weight. There was no significant difference in Smad3 expression level between control and db/db mice. Phosphorylation of the Smad3 C-terminal and linker domains was attenuated in *Smad3*^+/−^; db/db mice, compared with that in db/db mice. Therefore, Smad3 phosphorylation in diabetic glomeruli is an important event in DN progression.

We previously performed yeast one-hybrid assay to show that Smad1 transcriptionally regulated Col4 expression in DN^14^. Some studies have shown that the loss of *Smad3* expression attenuates diabetes-induced early glomerular changes in *Smad3*-knockout diabetic mice^35,36^. However, no study has assessed the interplay between Smad1 and Smad3 signaling under diabetic conditions so far. In the present study, we found that Smad3 expression considerably affected Smad1 activation, leading to ECM proteins overexpression in diabetic mice because Smad1 directly regulates ECM proteins expression^14,15^. Smad4 functions along with Smad1 and Smad3 as a common mediator of TGF-β signaling and performs various biological functions^37^. Therefore, reduced Smad3 signaling may relatively increase Smad1 activation. Our data indicate that attenuation of Smad3 signaling inactivates Smad1 by inhibiting the phosphorylation of its C-terminal domain and inducing the phosphorylation of its linker domain, which collectively lead to the regression of DN in *Smad3*^+/−^; db/db mice. This is because phosphorylation of the Smad1 linker domain inhibits the activity of Smad1^21,38^. These findings suggest that phosphorylation of the Smad1 linker domain or inhibition of the phosphorylation of the Smad1 C-terminal domain reduces ECM proteins expression in DN. Similar results were obtained *in vitro* by using the *Smad3*-null MCs and Smad3-over expressing MCs. All the data were obtained under a diabetic condition, indicating that TGF-β–Smad3 signaling modulates the phosphorylation of the Smad1 C-terminal and linker domains simultaneously in diabetes mellitus. Therefore, modulation of Smad3 expression levels and/or Smad3 phosphorylation may alter Smad1 activation, resulting in ECM proteins overproduction in DN.

In general, most patients with diabetes mellitus are treated with different kinds of drugs, resulting that diabetic patients with persistently high blood glucose level may be present in small number. Therefore, development of diabetic complications including DN has been known to be caused by exposure to AGE^25^. In this study, we compared the effects of exposure of high glucose and AGE on MCs, and we examined the expression and phosphorylation of Smad1 and Smad3. AGE stimulation showed significant activation of Smad1 and Smad3 in MCs compared with high glucose stimulation, which was consistent with our previous report^14^. In particular, phosphorylation of Smad1 was largely caused by AGE, suggesting that AGE has a critical role for the development of glomerulosclerosis through activation and modulation of Smad1 in MCs.

Protein glycation reactions leading to AGEs are thought to be the major causes of different diabetic complications^39^. Tissue and circulating AGE levels are known to be higher in diabetic patients and animals, which was consistent with our study. In particular, there is evidence that exposure to high levels of exogenous AGEs contributes to renal and vascular complications^40^. Thus, our study indicated that interplay between Smad1 and Smad3 has a major role in the development of DN in association with the exposure of AGE on MCs. Nevertheless, our experiments in this study indicates that a correlation between *in vitro* and *in vivo* observations still has some limitations that have to be considered in order to perform representative *in vitro* experiments that display there are some differences between *in vivo* studies.

Collectively, these results indicate that phosphorylation of the Smad1 linker domain is very important for attenuating DN. Level of pSmad1L increased in *Smad3*-null MCs but decreased in caSmad3-overexpressing MCs. Thus, pSmad1L levels are closely associated with pSmad3C levels in MCs. Based on these interactions between pSmad1L and pSmad3C, drugs that affect the interaction between pSmad1L and pSmad3C might serve as novel promising therapeutic agents. Probucol, a cholesterol-lowering drug, delayed DN progression in patients with type 2 diabetes in a randomized clinical study^41^. In the present study, we found that probucol-treated diabetic mice showed significant regression of DN. Moreover, probucol treatment enhanced the phosphorylation of the Smad1 linker domain. Probucol treatment also enhanced the phosphorylation of the Smad1 linker domain in BSA-treated MCs, suggesting that probucol exerted Smad3-independent effects on pSmad1L under non-diabetic condition. Similarly, probucol might exert additional beneficial effects on pSmad1L expression in a Smad3-independent manner in diabetic mice. A recent study suggested that probucol ameliorates diabetes-induced glomerulosclerosis by inhibiting expression of p66Shc which is mediator of mitochondrial ROS production^42^. However, the relationship between p66Shc and Smad1 is unclear and needs further investigation.

**Figure 9:**
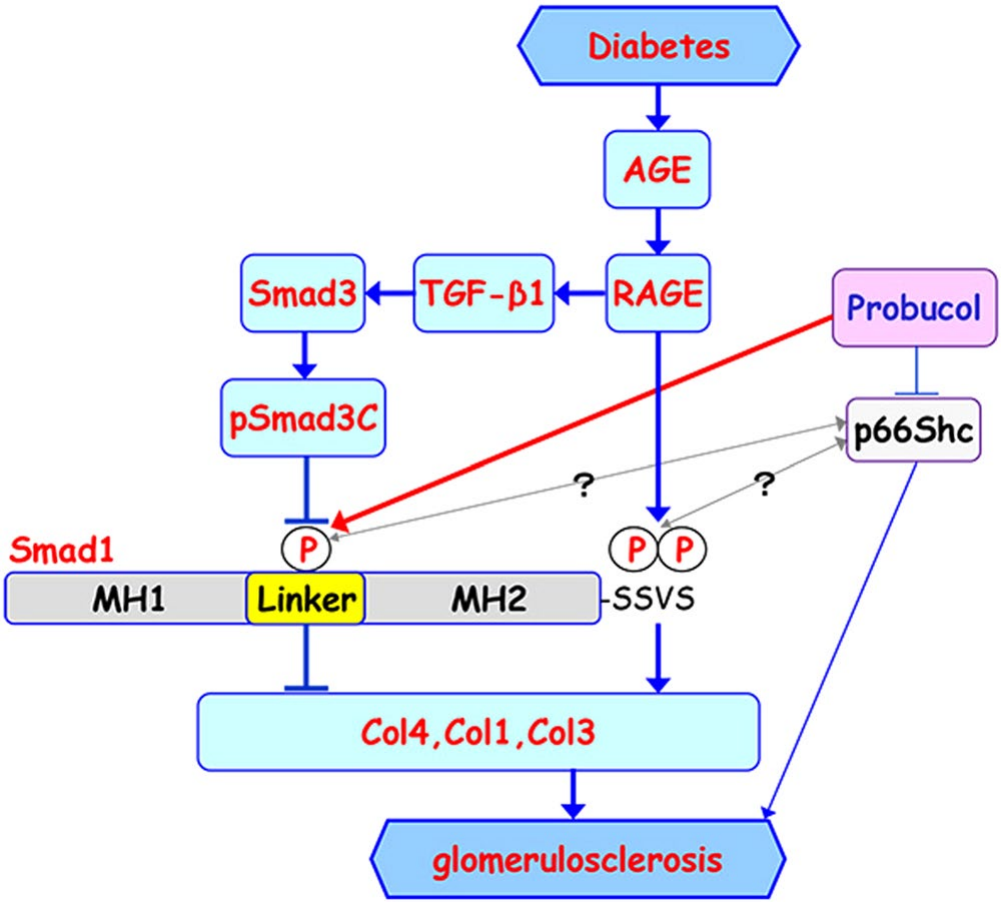
Proposed model for interplay between Smad1 and Smad3 in DN. In diabetes, phosphorylation of Smad1 at the SSVS motif in the C-terminal domain (pSmad1C) activated by AGE-RAGE axis induces excessive synthesis of ECM proteins (Col1, Col3, and Col4), leading to the development of glomerulosclerosis. In addition, TGF-β1 signaling pathway is also activated by AGE-RAGE axis and phosphorylates Smad3 (pSmad3C), which inhibits the phosphorylation of the Smad1 linker domain (pSmad1L). pSmad1L inhibits expression of Col1, Col3, and Col4, and thereby attenuates progression of glomerulosclerosis. Probucol may be involved in the activation of pSmad1L. Moreover, a recent report suggested that probucol ameliorates diabetes-induced glomerulosclerosis by inhibiting p66Shc expression, but the relationship between p66Shc and Smad1 is unknown. MH1 and MH2 stand for the N-terminal and C-terminal domains separated by a linker domain of Smad1, respectively.

In summary, we observed a novel interplay between Smad1 and Smad3 signaling under diabetic conditions and found that phosphorylation of the Smad1 linker domain may play a crucial role in DN progression (Fig. 9). Thus, preferential activation of the Smad1 linker domain may provide a novel therapeutic approach for treating DN without exerting undesirable adverse effects such as cancer, which is induced by the direct inhibition of Smad3^43,44^.

## Materials and Methods

### Animals

The animals were housed under specific pathogen-free conditions at the animal facility of Tokushima University. All animal experiments were performed in accordance with institutional guidelines, and the Review Board of Tokushima University granted ethical permission for this study. Six week old male C57BL/6 (WT), *BKS*/*Cg*-*m*^+/+^ Lepr db (db/db) and *BKS*/*Cg*-*m*^+/+^ Lepr db (db/+) mice were obtained from Charles River Japan (Tokyo, Japan). Heterozygous *Smad3*-knockout mice were kindly provided from Dr. Yasue, University of Tokushima. We tried to get *Smad3*-null mice using pairs of *Smad3*^+/−^ mice, but the fertility was low, and it was fragile and could not survive for the long term, at most 5 weeks. Therefore, *BKS*/*Cg*-*m*^+/+^ Lepr db (db/db) × *Smad3*^+/−^ mice were developed using pairs of Lepr db^+/−^ × *Smad3*^+/−^. *Smad3*^+/−^; db/+ mice were generated by crossing *Smad3*^+/−^ and db/+ mice. Moreover, *Smad3*^+/−^; db/db were developed by crossing *Smad3*^+/−^; db/+ and db/+ mice. All mice were genotyped by PCR. Confirmation of wild-type and null genotypes were performed by using PCR with primers: *Smad3*-S 5′-CCACTTCATTGCCATATGCCCTG-3′, *Smad3*-AS 5′-CCCGAACAGTTGGATTCACACA-3′, and neo-1 5′-CCAGACTGCCTTGGGAAAAGC-3′. The 6-week-old mice were classified into six groups as follows: (a) control diet C57BL/6 mice (n = 10); (b) control diet db/db mice (n = 10); (c) control diet *Smad3*^+/−^; db/db mice (n = 5); (d) 1% probucol diet (provided by Otsuka Pharmaceutical Co., Ltd.) C57BL/6 mice (n = 10); (e) 1% probucol diet db/db mice (n = 10); (f) 1% probucol diet *Smad3*^+/−^; db/db mice (n = 6). These mice were sacrificed at the end of 18 weeks after experiment, and their kidney weight and biochemical parameters were measured.

### Cell Culture Experiment

Glomerular MCs were established from glomeruli isolated from 4-week-old homozygous *Smad3*-knockout mice and their control littermates (strain background C57BL/6) and identified according to a method described previously^45^. MCs were maintained in B medium (a 3:1 mixture of minimal essential medium/F12 modified with trace elements) supplemented with 1 mM glutamine, penicillin at 100 units/ml, streptomycin at 100 mg/ml, and 10% fetal calf serum. The cultured cells fulfilled the generally accepted criteria for glomerular MCs^44^. MCs were treated with 0.1% dimethyl sulfoxide (DMSO) (vehicle) or probucol (30 μM) (provided by Otsuka Pharmaceutical Co., Ltd.).

### Preparation of AGEs

AGE-BSA was prepared by incubating BSA in phosphate-buffered saline (10 mM, pH 7.4) with 50 mM glucose 6-phosphate for 60 days at 37 °C as described previously^6,14^. Unmodified BSA was incubated under the same conditions without glucose 6-phosphate as control. Preparations were tested for endotoxin using an Endospecy ES-24S system (Seikagaku Co., Tokyo, Japan), and no endotoxin was detected. Protein concentration was measured by the Bradford method. All AGE-protein specific fluorescence intensities were measured at a protein concentration of 1 mg/ml. AGE-BSA and control BSA contained 51.3 and 2.74 units of AGE per milligram of protein, respectively.

### High glucose stimulation

The subconfluent MCs were cultured in serum-deprived media containing 5 mM D-glucose and 0.5% FBS for 48 h and then divided into three experimental groups according to glucose concentration. The normal glucose (NG) group comprised confluent cell monolayers cultured with 5 mM D-glucose, the high glucose (HG) group was cultured in 30 mM D-glucose and the osmotic control (OC) group was cultured in 30 mM D-mannitol.

### Plasmids

As previously described^28^, we obtained the full-length cDNAs of *Smad1* using gene-specific primers for reverse transcription-PCR (RT-PCR) and inserted into pcDNA3 resulting in pcDNA3-Smad1. To generate mammalian expression constructs of mutant versions of Smad1 (Smad1 (S206E) as a dominant positive and Smad1 (S206A) as a dominant negative), mutagenesis was carried out by a PCR-based approach by using QuickChange II Site-Directed Mutagenesis Kits (Agilent Technologies). The constructed plasmids were verified by sequencing. Moreover, constitutively active Smad3 (caSmad3) expression vector was kindly provided by Dr. J. Oh (Korea University).

### Electroporation

We electroporated (10 ms, 1500 V) MCs (1 × 10^6^ cells) cultured at 37 °C with 10 μg of plasmids with the Neon® Transfection System (Invitrogen). Cells were transferred to the 37 °C incubator and were cultured for further experiments.

### Biochemical Parameters and Urine Examination

Urinary albumin excretion was measured at 0, 4, 9, 13, and 17 weeks after 1% probucol or control diet were given. Twenty four-hour urine collection samples from mice housed in individual metabolic cages were measured. During the urine collection, the mice were allowed free access to food and water. Albumin concentration in the urine was measured using the Albuwell kit (Exocell Inc., PA). Creatinine concentration in the urine was measured using LabAssay TM Creatinine (290–65901; Wako). Levels of serum creatinine (Cr), blood urea nitrogen (BUN), total cholesterol (TC), low density lipoprotein cholesterol (LDL-C) and high density lipoprotein cholesterol (HDL-C) were measured at 18 weeks. Hemoglobin A1c was measured before starting experiment and at 18 weeks.

### Western Blotting

Western blot analysis was performed as we previously described^46,47^. Briefly, cell lysates or proteins from mouse renal cortex were extracted and probed with anti-Smad3, anti-Col1 alpha 2 chain, anti-GAPDH, anti-TGF-β1, anti-TGF-β2 and anti-p-p65 (Abcam), anti-pSmad3C and anti-pSmad1C (Cell Signaling Technology), anti-pSmad3L (IBL), anti-Smad1 (Bio Matrix Research), anti-pSmad1L (S206) (SAB), anti Col4 alpha 1 and 2 chains (Southern Biotech), anti-TGF-β3 (R&D Systems), anti-RAGE (GeneTex), and anti-p65 (Santa Cruz) antibodies, followed by incubation with horseradish peroxidase-conjugated secondary antibody (GE Healthcare). The immunoreactive bands were visualized using an ECL Western blotting detection system (GE Healthcare). In the process of development of glomerulosclerosis, glomerular mesangial cells differentiate into myofibroblasts that are responsible for the synthesis and accumulation of interstitial ECM components such as type I and III collagens, leading to glomerulosclerosis. In addition, the myofibroblast has gene expression signatures showing similarities to pericytes, muscle cells, endothelial cells, neurons, and phagocytes^48^. Moreover, in diabetic nephropathy, as glomerulosclerosis develops, exact identification of mesangial cells becomes difficult due to extracellular matrix (ECM) accumulation and subsequent destruction of glomerular architecture. Therefore, it is hard to precisely measure the number of mesangial cells in diabetic glomeruli. Hence, we performed the immunoblotting analyses of renal cortex lysates from each group and we assessed quantitative relationships between Smads.

### Measurement of the AGE levels in serum and kidney

Concentrations of AGE-modified plasma and renal proteins were determined by measurement of fluorescence using an excitation wavelength of 340 nm and an emission wavelength of 415 nm as described elsewhere^29^. Measurement of protein concentration in plasma and in extracts of the renal cortex allowed expression of the data as arbitrary fluorescence units normalized to protein content.

### Histological examination

Tissue for light microscopy was fixed in methyl Carnoy’s solution and embedded in paraffin. Sections (2 μm thick) were stained with periodic acid Schiff (PAS) and periodic acid methenamine silver (PAM) as we previously described^49^.

### Immunohistochemistry

Cryopreserved kidney tissues were cut in 4-μm-thick sections and fixed in methanol at 4 °C for 15 min. To eliminate nonspecific staining, sections were incubated with the appropriate preimmune serum for 30 min at room temperature, followed by incubation with primary antibodies, anti-Smad1 (Bio Matrix Research), anti-pSmad1C and anti-pSmad3C (Cell Signaling Technology), anti-pSmad1L (S206) (SAB), anti-pSmad3L (IBL), anti-Smad3 and anti-Col1 alpha 2 chain (Abcam), anti Col4 alpha 1 and 2 chains (Southern Biotech), and anti-Col3 alpha 1 chain (Santa Cruz) antibodies followed by incubation with the appropriate fluorescent secondary antibodies. Staining with DAPI was performed to identify the nuclei of cells.

### Quantitation of Light Microscopy

Glomerular morphometry was evaluated in PAM-stained tissues. The glomerular surface area and the PAM-positive area/glomerular area (%) were measured using Image J (NIH). For each animal, all glomeruli were analyzed.

### Quantitation of Immunohistochemistry

To examine localization of pSmad1C, pSmad1L, pSmad3C, and pSmad3L, a blind test evaluated 20 average size of glomeruli in each specimen. The number of positive nuclei (merge of red and blue) was counted in each phosphorylated Smad protein, and the mean values were calculated. Expression of Smad1, Smad3, Col1, and Col3 were assessed by quantitative PCR analysis.

### Quantitative RT-PCR analysis

Total RNA was extracted from renal cortex using the Pico Pure RNA Isolation Kit (Molecular Devices, CA) according to the manufacturer’s protocol. The RT-PCR assays were performed at least three times in triplicate. Reverse transcription from mRNA to cDNA was performed using SuperScript reverse transcription kits (Invitrogen, CA). Amplification was conducted in MiniOpticon real-time PCR detection system (Bio Rad) using TaqMan gene expression assays (Applied Biosystems, CA). The cycling parameters were 10 minutes at 95 °C, followed by 50 cycles of 15 sec at 95 °C and 60 sec at 60 °C. Normalization was performed using Rn18s as internal standards.

### Statistical analysis

All data are presented as the mean ± S.E. Unpaired Student’s t test for comparison between 2 groups were performed. P values < 0.05 were considered to be statistically significant. Densitometric analysis in Western Blotting was performed using NIH Image J software.

## Electronic supplementary material

Supplementary Information
