# Supplementary material for: Novel Interplay Between Smad1 and Smad3 Phosphorylation via AGE Regulates the Progression of Diabetic Nephropathy
Source: Sci Rep. 2018 Jul 12;8:10548. doi: 10.1038/s41598-018-28439-1 (PMC6043613; doi:10.1038/s41598-018-28439-1)
Supplement: Supplementary file 1 — Supplementary Information [file 41598_2018_28439_MOESM1_ESM.pdf]

Supplementary Information

Novel Interplay Between Smad1 and Smad3 Phosphorylation via AGE Regulates the Progression of Diabetic Nephropathy

Hiroyuki Ono[1], Hideharu Abe[1*], Akiko Sakurai[1], Arisa Ochi[1], Tatsuya Tominaga[1], Masanori Tamaki[1], Seiji Kishi[1], Taichi Murakami[1], Kojiro Nagai[1], Masayuki Kohashi[2], and Toshio Doi[1]

[1]Department of Nephrology, Institute of Biomedical Sciences, Tokushima University Graduate School, Tokushima, Japan, [2]Biology and Translational Research Unit, Department of Medical Innovations, New Drug Research Division, Otsuka Pharmaceutical. Co. Ltd., Tokushima, Japan

*Correspondence should be addressed to Hideharu Abe, M. D., Ph. D.

Department of Nephrology, Institute of Health Biosciences, University of Tokushima Graduate School, Tokushima, 770-8503, Japan

Tel: 81-88-633-7184, Fax: 81-88-633-9245,

E-mail: abeabe@tokushima-u.ac.jp.

List of Materials

Supplementary Figure S1

Supplementary Figure S2

Supplementary Figure S3

Supplementary Figure S4

Supplementary Figure S5

Supplementary Figure S6

Supplementary Figure S7

Supplementary Figure S8

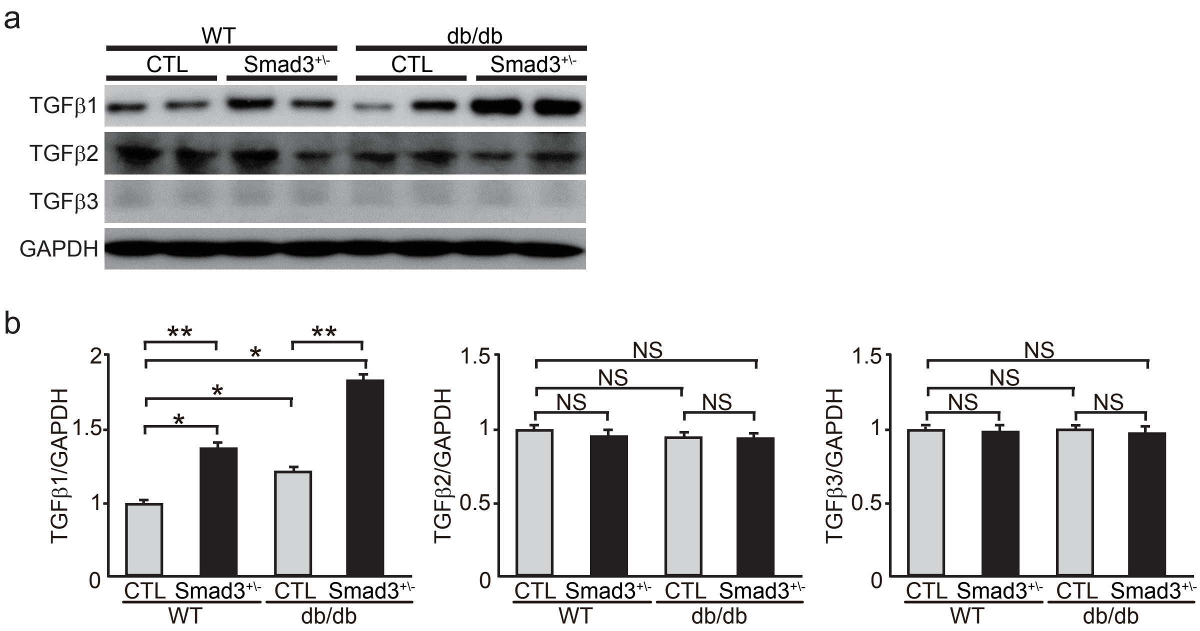

Supplementary Figure S1. Expression of TGF-β isoforms in diabetic Smad3 knockout mice. (a) Western blot analysis for TGF-β isoforms in protein obtained from cortical tissue of kidneys. Equal amounts of cell lysates were subjected to western blotting. GAPDH was used as a loading control. One of three independent experiments is shown. **(b)** Optical densitometry of these proteins in western blot was shown. Data represent mean values ± S.E. of at least three independent experiments; n = 10 for normal control mice, n = 10 for *Smad3*$^{+/-}$ mice, n = 10 for db/db mice, n = 5 for *Smad3*$^{+/-}$;db/db mice. NS, not significant, *, $p < 0.05$ versus normal control mice, **, $p < 0.05$ versus *Smad3* wild-type mice, t test). WT and CTL stand for nondiabetic mice and *Smad3* wild-type mice, respectively.

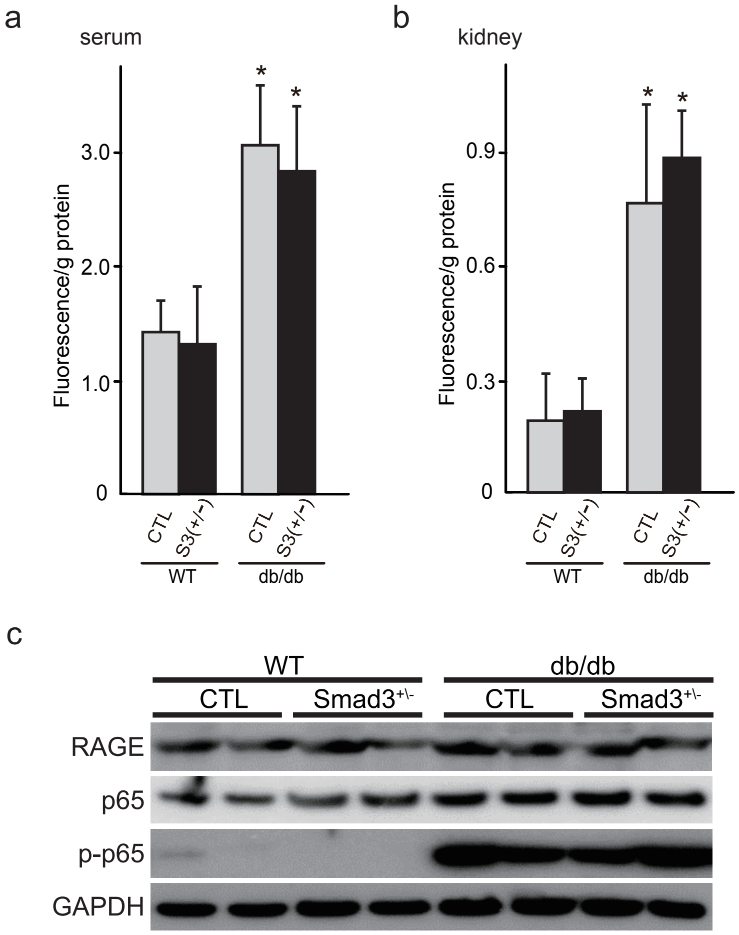

Supplementary Figure S2. Expression and activation of AGE-RAGE-NFκB signal in diabetic mice. Fluorescence of serum **(a)** and kidney **(b)** proteins. Results expressed as arbitrary fluorescence untis/mg protein and represent mean ± S.E. of measurements in each group of mice. **(c)** Western blot analysis for RAGE and NFκB subunit p65 in protein obtained from cortical tissue of kidneys. Equal amounts of cell lysates were subjected to western blotting. GAPDH was used as a loading control. One of three independent experiments is shown. WT and CTL stand for nondiabetic mice and *Smad3* wild-type mice, respectively.

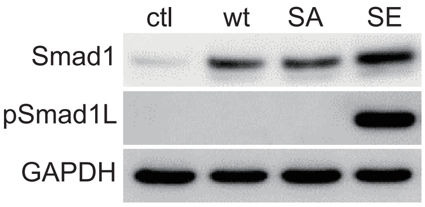

Supplementary Figure S3. Phosphorylation of Smad1 linker region in MCs transfected with various constructs. MCs are harvested 48 h post-transfection with plasmids and equal amounts of cell lysates were subjected to Western blotting. GAPDH was used as a loading control. One of three independent experiments is shown. ctl, empty vector; wt, wild-type Smad1; SA, Smad1 carrying S206A mutation; SE, Smad1 carrying S206E mutation.

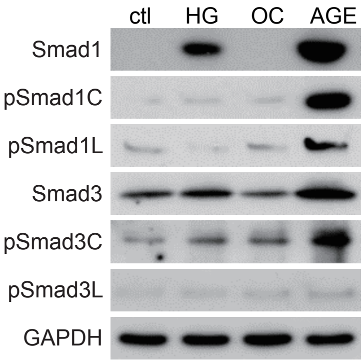

Supplementary Figure S4. Expression and activation of Smad1 and Smad3 in MCs in diabetic conditions. MCs were cultured in medium containing normal glucose (ctl, 5 mM), high glucose (HG, 30 mM), D-mannitol (30 mM) (OC) or AGE (5μg/ml) for 48 h. Equal amounts of cell lysates were subjected to western blotting. GAPDH was used as a loading control. One of three independent experiments is shown.

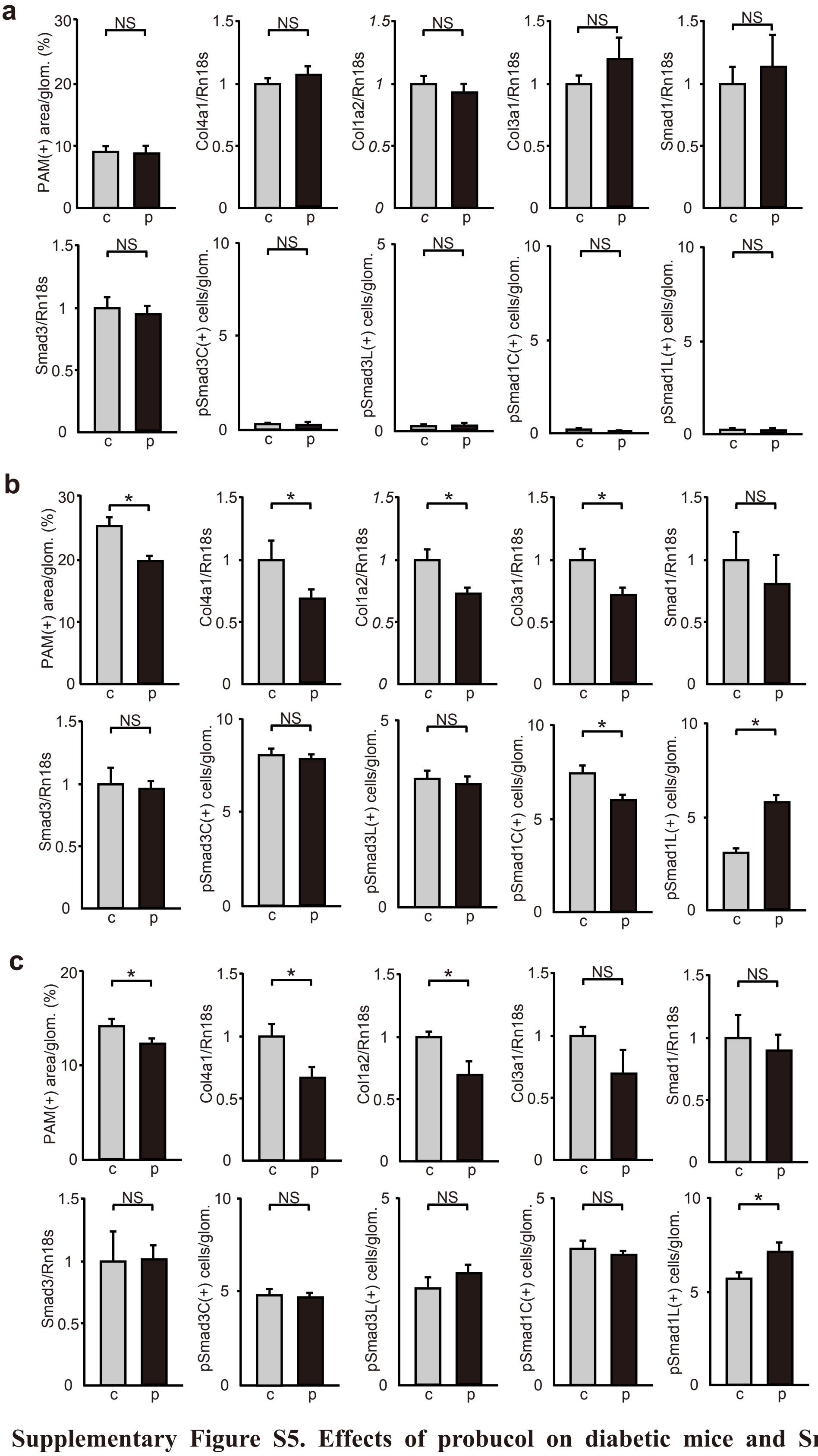

Supplementary Figure S5. Effects of probucol on diabetic mice and Smad3 knockout diabetic mice. Quantitation of Col4, Col1, Col3, Smad1, and Smad3 were performed by qPCR and increment of phosphorylation regarding Smad1 and Smad3 were assessed by counting positive glomerular nuclei between untreated normal control mice and probucol-treated normal contorl mice **(a)**, between untreated db/db mice and probucol-treated db/db mice **(b)**, and between untreated *Smad3*$^{+/-}$;db/db mice and probucol-treated *Smad3*$^{+/-}$;db/db mice **(c)**. Data obtained by qPCR were normalized to the expression of Rn18s. As to the number of positive glomerular nuclei, the mean values (per one glomerulus) were calculated. Moreover, mesangial sclerotic fraction in the above two groups was determined as percentage of mesangial matrix area per total glomerular surface area. All glomeruli were analyzed for each sample. Results are expressed as the mean ± S.E. (NS, not significant, *, $p < 0.01$ versus untreated db/db mice, or versus untreated *Smad3*$^{+/-}$;db/db mice, t test). C and p stand for control diet- and probucol diet-treated mice, respectively.

Figure 3c

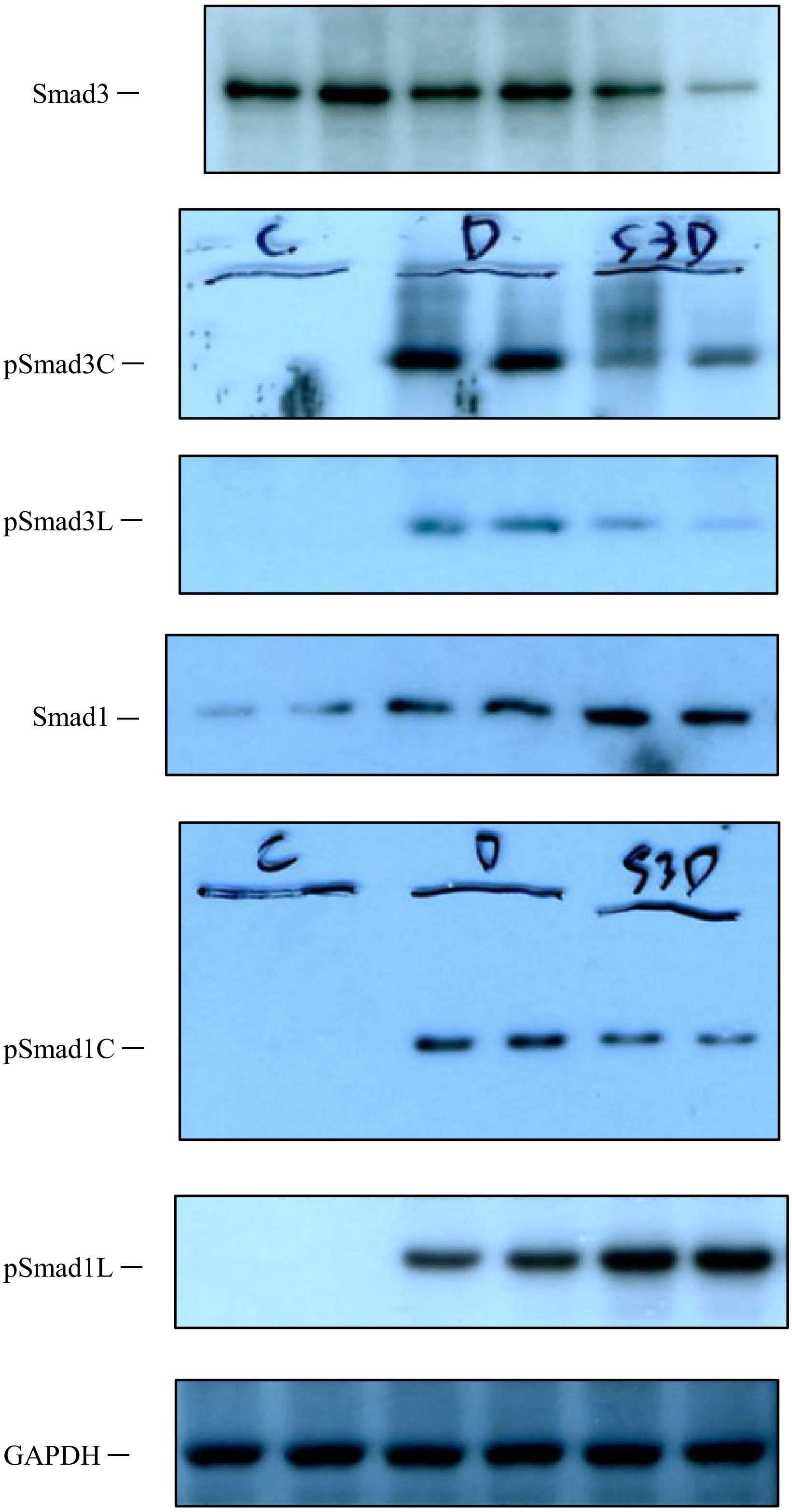

Supplementary Figure S6. Uncropped scans of blots (Figure 3c).

Figure 4a

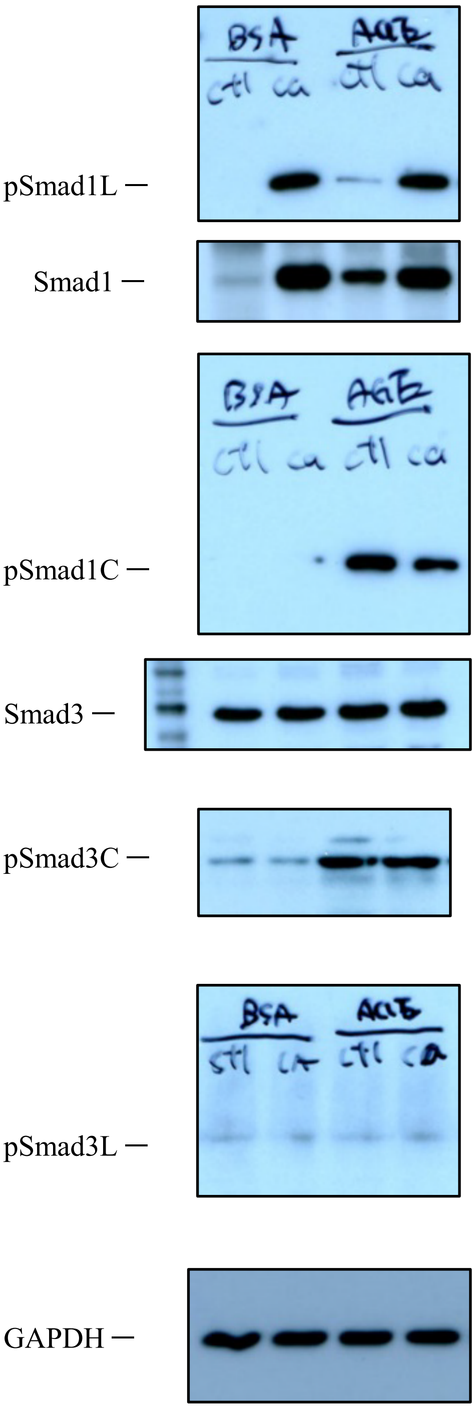

Figure 4c

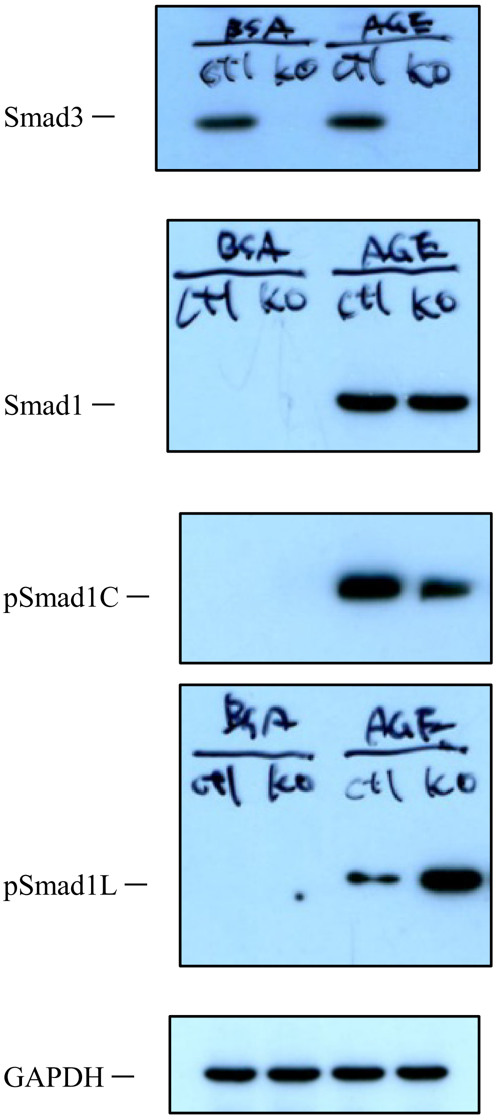

Supplementary Figure S7. Uncropped scans of blots (Figure 4a and Figure 4c).

Figure 5a

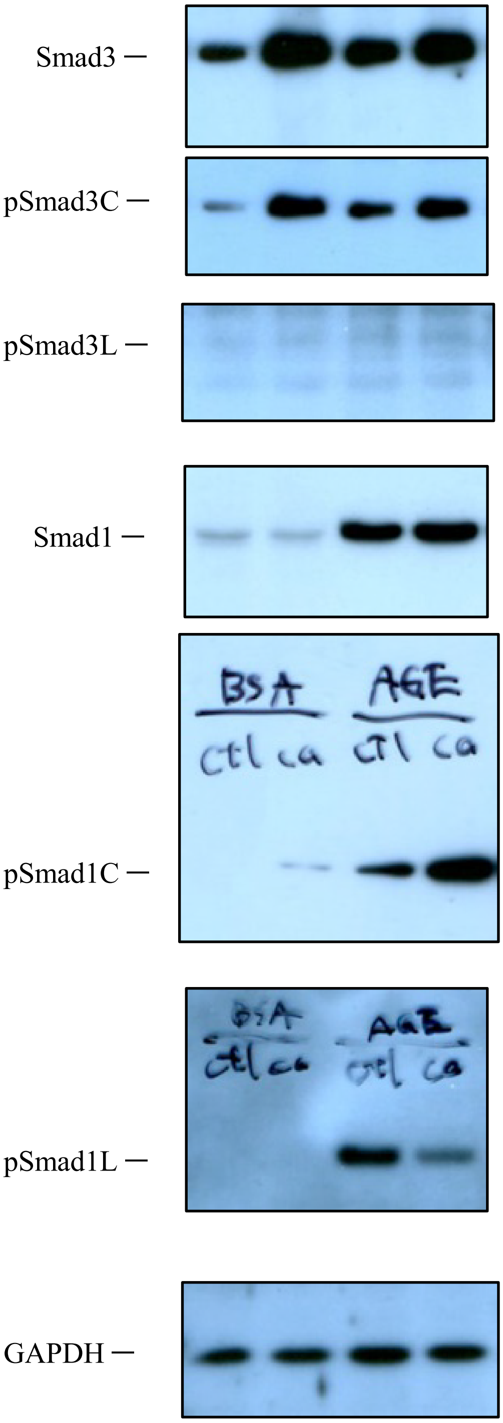

Figure 6a

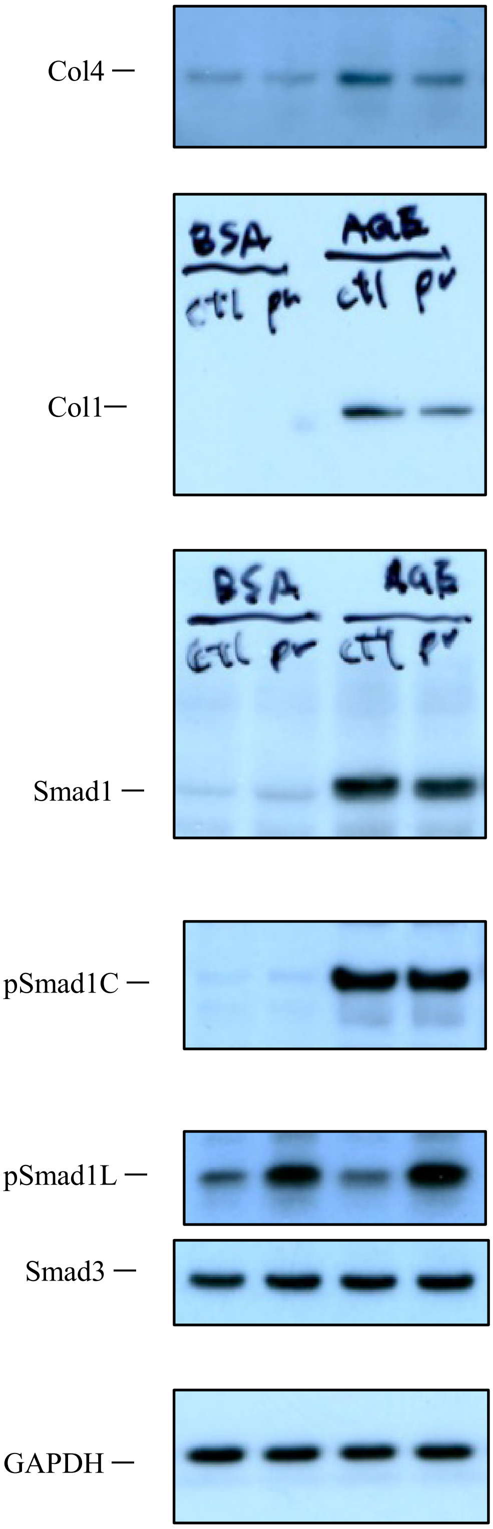

Supplementary Figure S8. Uncropped scans of blots (Figure 5a and Figure 6a).
